# Effect of Yellowing Duration on the Chemical Profile of Yellow Tea and the Associations with Sensory Traits

**DOI:** 10.3390/molecules27030940

**Published:** 2022-01-29

**Authors:** Fang-Yuan Fan, Sen-Jie Zhou, Hong Qian, Bang-Zheng Zong, Chuang-Sheng Huang, Ruo-Lan Zhu, Hao-Wei Guo, Shu-Ying Gong

**Affiliations:** 1 Tea Research Institute, Zhejiang University, 866 Yuhangtang Road, Hangzhou 310058, China; FangyFan@zju.edu.cn (F.-Y.F.); zhousenjie@zju.edu.cn (S.-J.Z.); myzone@zju.edu.cn (B.-Z.Z.); 21816162@zju.edu.cn (C.-S.H.); 22016172@zju.edu.cn (R.-L.Z.); hwguo@zju.edu.cn (H.-W.G.); 2 Deqing Agricultural Technology Extension Center, 883 Zhongxingbei Road, Huzhou 313200, China; tannin@126.com

**Keywords:** yellow tea, yellowing, sensory traits, volatiles, non-volatiles, multiple factor analysis

## Abstract

The yellowing process is the crucial step to form the characteristic sensory and chemical properties of yellow tea. To investigate the chemical changes and the associations with sensory traits during yellowing, yellow teas with different yellowing times (0–13 h) were prepared for sensory evaluation and chemical analysis. The intensities of umami and green-tea aroma were reduced whereas sweet taste, mellow taste and sweet aroma were increased under long-term yellowing treatment. A total of 230 chemical constituents were determined, among which 25 non-volatiles and 42 volatiles were the key chemical contributors to sensory traits based on orthogonal partial least squares discrimination analysis (OPLS-DA), multiple factor analysis (MFA) and multidimensional alignment (MDA) analysis. The decrease in catechins, flavonol glycosides and caffeine and the increase in certain amino acids contributed to the elevated sweet taste and mellow taste. The sweet, woody and herbal odorants and the fermented and fatty odorants were the key contributors to the characteristic sensory feature of yellow tea with sweet aroma and over-oxidation aroma, including 7 ketones, 5 alcohols, 1 aldehyde, 5 acids, 4 esters, 5 hydrocarbons, 1 phenolic compound and 1 sulfocompound. This study reveals the sensory trait-related chemical changes in the yellowing process of tea, which provides a theoretical basis for the optimization of the yellowing process and quality control of yellow tea.

## 1. Introduction

Yellow tea is a special type of tea characterized with the yellow color of dry tea, tea infusion and infused leaves, as well as mellow and smooth taste [1]. Yellow tea has a long history in China, which draws increasing attention of consumers due to its unique color, less bitter and astringent flavor. It is also associated with many biological activities, such as antioxidant and antimicrobial activities, and improvement in lipid metabolism [1]. In China, the production yield of yellow tea has grown by more than 300% in the past five years according to the report of China Tea Marketing Association, implying a rapid expansion market of yellow tea. The characteristic flavor of yellow tea is attributed to the taste-related chemical composition of tea leaves, such as caffeine, catechins, amino acids, flavone and soluble sugar [2,3], as well as the aroma-related chemical composition of yellow teas, e.g., alcohols, aldehydes, hydrocarbon, ketones and ethers [1,4].

The unique flavor of yellow tea forms mainly due to the special processing step in the production of yellow tea, namely the “yellowing” step, which is the most important step different from the production of green tea [1]. During “yellowing”, moisture-dependent thermochemical reactions occur, leading to the formation of yellow tea leaf color and unique flavor [5]. Specifically, tea leaves turn yellow and then the color is gradually deepened [6], accompanied by a decrease in bitterness and astringency whereas an increase in sweetness [5]. Polyphenols are the major bioactive and sensory quality-related components of yellow tea. The yellowing process causes an increase in catechins (C), gallocatechin gallate (GCG) and theaflavins whereas a decline in epigallocatechin-3-gallate (EGCG) and the ratio of tea polyphenols to amino acids [1,7,8]. Moreover, the content of gallic acid in yellow tea is more than 10-fold greater than green tea, suggesting the important role of gallic acid in constructing the taste of yellow tea [1,5]. Wei et al. [6] reported that the levels of most catechins, amino acids, phenolic acids and glycosyl derivatives of volatiles decreased significantly, whereas betaine, piperidine, theasinensin B and lysophosphatidylcholines increased significantly after 24 h of yellowing. Among these constituents, the contents of most amino acids and sucrose were positively related with the intensities of umami and sweetness, the contents of catechins and phenolic acids were positively correlated with the intensities of bitterness and astringency, while phaeophorbides had a positive correlation with red color of tea leaves [6]. Moisture content, temperature, duration and ventilation frequency are the key factors affecting the chemical conversions in the yellowing process [9]. The lower water content caused by pre-drying before yellowing could lead to the accumulation of free amino acids and soluble sugars, and an increase in the brightness and yellow color of tea infusion [5]. The ventilation frequency and relative humidity impacted the contents of catechins (CG, ECG, GCG), flavonoid glycosides (myricetin rhamnoside, quercetin galactoside) and partial amino acids (theanine, serine, glutamine, arginine, histidine, γ-aminobutyric acid) in yellow tea [9]. 

The chemical changes of yellow tea during the yellowing process have been investigated, mainly focusing on the non-volatile compounds related to taste [1,5,6]. Sensory flavor is a combination of taste and smell, which is related to the compositions of non-volatiles and volatile compounds, especially the volatiles involved in retronasal identification [10]. Thus, the chemical transformations of both volatile and non-volatile compounds during yellowing and their correlations with sensory feature changes need overall investigations. In this study, we investigated the flavor changes and the chemical conversions of non-volatiles/volatiles in tea leaves during yellowing, which provided a theoretical foundation for further optimization of the yellowing process. The sensory evaluation of yellow tea was carried out by quantitative descriptive analysis (QDA), and the non-volatile compounds in yellow tea were analyzed by high-performance liquid chromatography (HPLC) and ultra-high-performance liquid chromatography–mass spectrometry (UPLC-MS/MS), while the volatile compounds were analyzed by gas chromatography–mass spectrometry (GC-MS). Orthogonal partial least squares discrimination analysis (OPLS-DA) was used to explore the potential correlations between flavor traits and chemical compositions. The key contributors to flavor traits were screened out by multiple factor analysis (MFA) and multidimensional alignment (MDA). 

## 2. Results

### 2.1. Sensory Analysis of Yellow Tea

**Figure 1:**
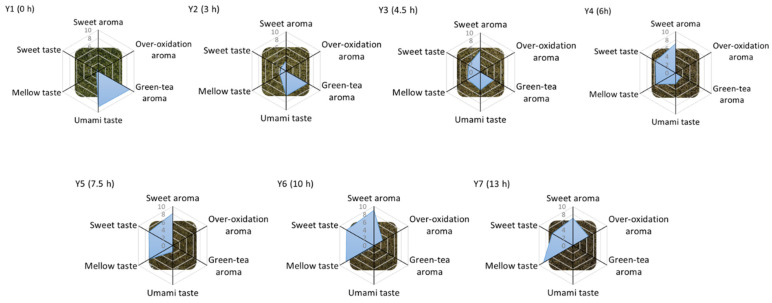
Sensory properties of yellow teas with different yellowing times.

Figure 1 shows the flavor changes of yellow teas under different yellowing treatments. Dry tea gradually turned from green to yellow and then to brown as yellowing time increased, which was in an agreement with the previous study [6]. Six sensory attributes of samples were evaluated (Figure 1 and Table S1), including three taste attributes (umami, mellow and sweet taste) and three aroma attributes (sweet aroma, over-oxidation aroma and green-tea aroma). The tea sample (Y1) without the yellowing process (time = 0 h) was characterized by umami taste (intensity score = 8.67) and green-tea aroma (intensity score = 8.83). With an increase in yellowing time, umami taste and green-tea aroma were decreased, while mellow taste, sweet taste and sweet aroma were increased. The highest intensity of soft taste was achieved in sample Y7 (8.67). Sample Y6 under 10 h yellowing treatment had the highest intensity of sweet taste (8.00) and sweet aroma (9.00) in this study. Both decreased significantly when yellowing time exceeded 10 h. However, the oxidized aroma was induced after long-term yellowing treatment. 

### 2.2. Chemical Changes of Flavor Compounds under Yellowing

**Figure 2:**
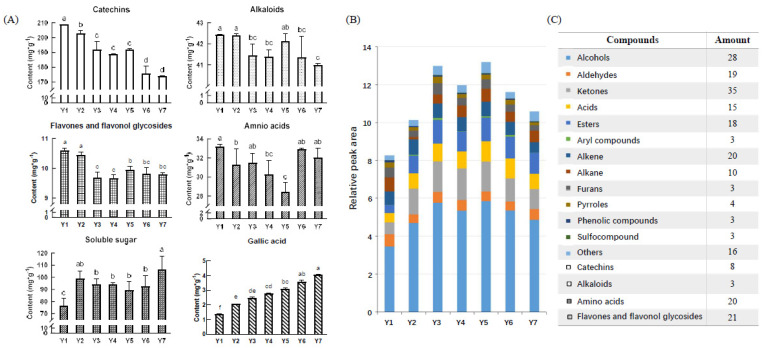
Comparison of non-volatiles and volatiles in yellow teas with different yellowing times. (**A**) Non-volatiles; (**B**) volatiles; (**C**) compounds and corresponding amounts. Values (**A** & **B**) are given as mean (n = 3). Means with different letters (**A**) are significantly different from one another according to a one-way ANOVA.

Figure 2 shows the chemical compositions of yellow teas under different yellowing treatments. A total of 230 flavor-related chemical compounds/indexes were detected in 21 yellow tea samples, including total soluble sugar, 8 catechins, 21 flavones and flavonol glycosides, 3 alkaloids, 20 amino acids, and volatile compounds (28 alcohols, 19 aldehydes, 35 ketones, 15 acids, 18 esters, 3 aryl-compounds, 20 alkenes, 10 alkanes, 3 furans, 4 pyrolysis, 3 phenolic compounds, 3 sulfo-compounds and 16 others). Catechins, flavones and flavonol glycosides, alkaloids, amino acids and soluble sugar were taste-related substances, among which catechins had the highest contents (Figure 2A). The sample Y1 had the highest levels of catechins (208.90 mg g^−1^), flavones and flavonol glycosides (10.59 mg g^−1^), alkaloids (42.43 mg g^−1^) and amino acids (33.21 mg g^−1^). The contents of catechins gradually decreased with yellowing duration, achieving the lowest level after 13 h yellowing treatment. The content of total amino acids slightly declined during the first 7.5 h but increased afterwards, and the sample Y5 had the lowest content of total amino acids at 28.45 mg g^−1^. The levels of total soluble sugars in the samples (Y2-Y7) under 3–13 h yellowing treatments were significantly higher than Y1 without yellowing treatment, but no obvious change trend was observed for the total soluble sugars during yellowing. Figure 2B showed the composition of volatile compounds in different yellow teas. Total peak areas ratio (PAR) of volatiles increased during the first 7.5 h yellowing and then significantly declined as yellowing time exceeded 10 h. Alcohols were the dominant volatile type, followed by ketones and esters, and these three types of volatiles showed the same change tendency of being increased initially and then decreased slightly. The composition of volatiles was in line with findings of the previous study that alcohols and esters were the major volatiles in yellow teas accounting for 26.30% and 26.14% of the total volatiles [4]. The PAR of alcohols, ketones, acids and esters increased during yellowing.

### 2.3. OPLS-DA of Flavor Compounds in the Yellowing Process

**Figure 3:**
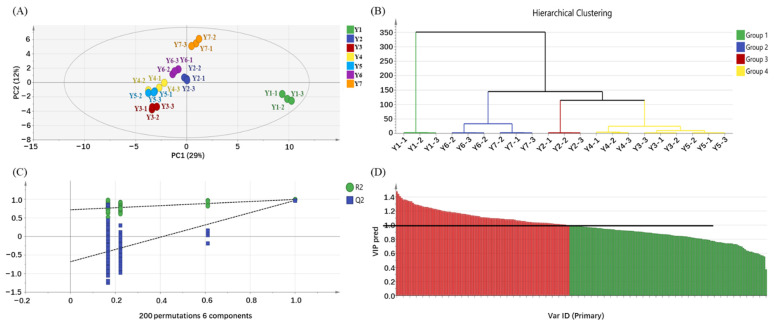
Multivariate analysis of yellow tea yellowing process duration. (**A**) OPLS-DA score plot, R^2^X = 0.814, R^2^Y = 0.981, Q^2^ = 0.849. (**B**) Dendrogram plot of yellow teas. (**C**) Permutation plot of OPLS-DA, R^2^ = 0.722, Q^2^ = −0.694. (**D**) VIP plot of flavor–chemical constituents.

OPLS-DA was used for discriminating samples based on the chemical composition. The OPLS-DA model had a good predictive power (R^2^X = 0.814, R^2^Y = 0.981, Q^2^ = 0.849, Figure 3A) and cross-validation (intercepts of R^2^ = 0.722 and Q^2^ = −0.694, Figure 3C), with the *p*-value being 2.82e-006. Both the score plot (Figure 3A) and hierarchical cluster analysis (HCA, Figure 3B) showed that different yellow teas were discriminated from each other based on the chemical compositions. Tea sample without yellowing (Y1) was clearly isolated from other yellow tea samples in the direction of PC1, while yellow teas samples (Y2–Y7) were discriminated from each other in the direction of PC2. The values of variable importance in the projection (VIP) were used for screening key chemical constituents. There were 98 key chemical contributors screened out according to the criterion of VIP > 1.0 and *p* < 0.05 (Figure 3D), including 40 non-volatiles and 67 volatiles. Particularly, kaempferol-3-*O*-glucoside-rhamnoside-rhamnoside, methyl jasmonate and 3,8-dimethyl-decane had higher VIP values (>1.4), which were regarded as the key compounds of the quality of yellow tea. 

### 2.4. Association between Key Chemical Contributors and Sensory Traits

**Figure 4:**
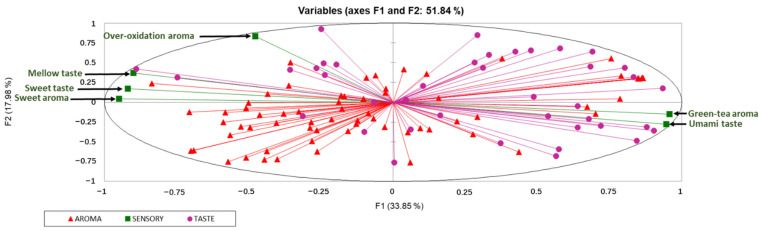
MFA loading plot of sensory traits and key chemical contributors.

**Figure 5:**
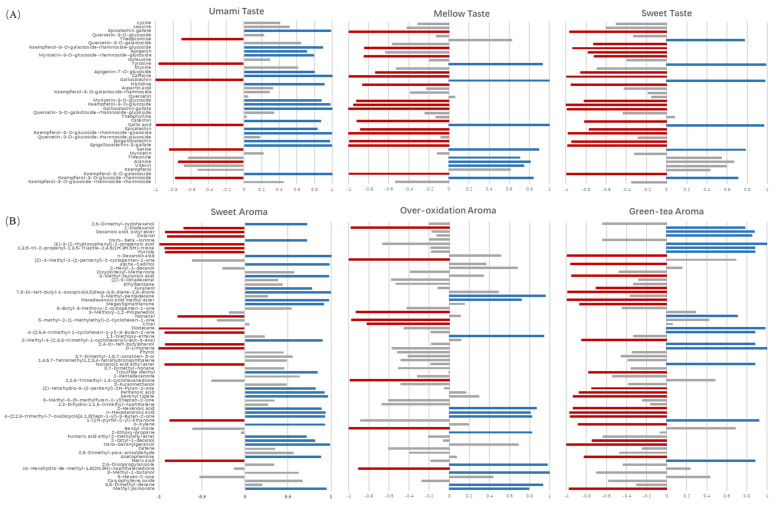
Associations between sensory traits and key chemical contributors based on MFA. (**A**) Taste traits; (**B**) aroma traits.

**Figure 6:**
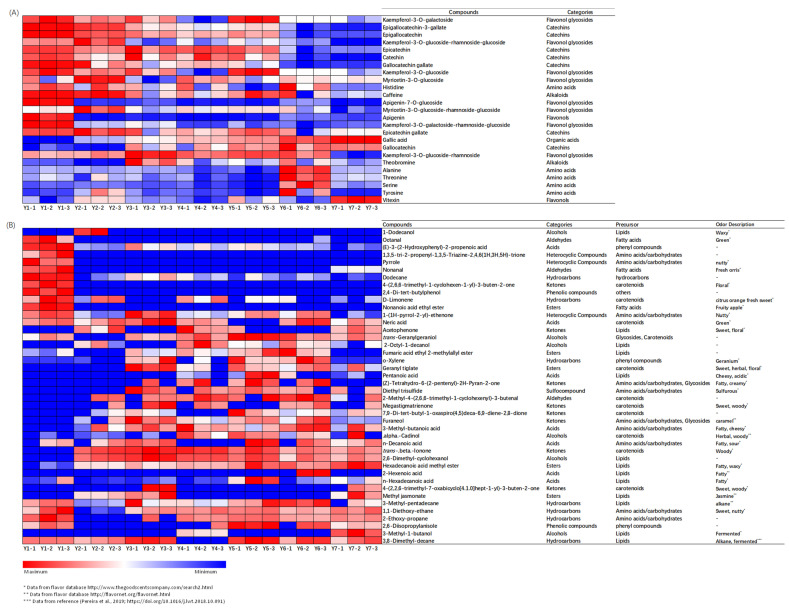
Heatmap of key chemical contributors to sensory traits in yellow teas with different yellowing times. (**A**) Non-volatiles; (**B**) volatiles.

Multiple factor analysis (MFA) was used to explore the correlations between sensory traits and key chemical constituents (VIP > 1.0 based on OPLS-DA) (Figure 4). The first two factors (51.84% of total variance) were selected for the association analysis. The loading plot of MFA showed that green-tea aroma and umami traits were distributed at the positive end of F1, while sweet taste, mellow taste, sweet aroma and over-oxidation aroma traits were distributed at the negative end of F1. The association between sensory traits and key chemical compounds were further assessed by multidimensional alignment (MDA) based on MFA. When the cosine value is below +/−0.707, no correlation is considered. As shown in Figure 5A, the contents of kaempferol-3-*O*-glucoside-rhamnoside, serine, gallic acid, gallocatechin and tyrosine were positively related with sweet taste and mellow taste, while were negatively related with umami taste. In addition, threonine, alanine and vitexin were associated with mellow taste with cosine values of 0.71, 0.81 and 0.75, respectively. The sweet taste-related and mellow-related compounds in yellow teas increased as the yellowing duration increased, achieving the highest level during 10–13 h (Figure 6A). Sample Y1 contained the highest content of umami-related constituents mainly including catechins, partial flavonoids (Figure 6A). 

There were 13 volatiles significantly correlated with green-tea aroma, the levels of which drastically decreased with yellowing duration (Figure 6B). Most of the volatiles were characterized by green, fresh floral, fresh sweet or fresh fruity, except pyrrole and 1-(1H-pyrrol-2-yl)-ethanone with nutty-like odor (Figure 6B), which was consistent with the typical fresh and chestnut-like odor of green tea [11,12,13]. In Figure 5B, there were 6 ketones, 5 acids, 4 alcohols, 4 esters, 1 hydrocarbons and 1 aldehyde associated with sweet aroma, among which methyl jasmonate, 4-(2,2,6-trimethyl-7-oxabicyclo[4.1.0]hept-1-yl)-3-buten-2-one, n-hexadecanoic acid, 2-hexenoic acid and hexadecanoic acid methyl ester were also significantly related with over-oxidation aroma. In addition, other six volatiles with high cosine values in Figure 5B were considered as the key chemical contributors to the over-oxidation aroma, including 3,8-dimethyl-decane (0.932), 3-methyl-1-butanol (0.995), 2,6-diisopropylanisole (0.974), 2-ethoxy-propane (0.819), 1,1-diethoxyethane (0.942) and 3-methyl-pentadecane (0.955). Most of the volatiles associated with sweet aroma were characterized with sweet, herbal and woody odors, while the volatiles associated with over-oxidation aroma were characterized with fatty and fermented odors (Figure 6B). Higher levels of sweet aroma-related volatiles were detected in the samples of Y3, Y4, Y5 and Y6 under 4.5–10 h yellowing treatments, while the higher levels of over-oxidation aroma-related volatiles were observed in sample Y7 under 13 h yellowing treatment (Figure 6B). 

## 3. Discussion

The sensory quality of yellow tea is associated with chemical composition, which is greatly impacted by the yellowing process. In our study, the levels of catechins, flavones and flavonol glycosides generally declined with yellowing duration, while the content of gallic acid gently increased. This is consistent with a previous study that found that long-term yellowing reduced the contents of catechins as well as the intensities of bitterness and astringency [6]. Gallic acid is an important hydrolysis product of gallated catechins [14], which was increased with yellowing duration. Catechins, flavones and flavonol glycosides are important contributors to the bitterness and astringency of teas [15], which were susceptible to high temperature due to degradation, oxidation and hydrolysis reactions [1,14,16]. Thus, oxidation/polymerization may also occur in flavonoid compounds in addition to hydrolysis reactions. Amino acids are important umami and sweet substances in tea infusion [15]. Free amino acids were increased after yellowing, which was attributed to the hydrolysis of proteins as well as the derivatization of certain amino acids, such as betaine and pipecolic acid isomers [6]. Our study showed that the intensity of umami taste was apparently associated with the contents of catechins, caffeine, flavones and flavonol glycosides. That is because umami taste was weakened by yellowing, meanwhile the contents of catechins, flavonol glycosides and caffeine were also decreased coincidently. Sweet-tasting amino acids were increased during yellowing, such as alanine, threonine, serine and tyrosine [15], which might contribute to the mellow taste of tea infusion. The reduced bitter and astringent compounds (e.g., catechins, flavones and flavonol glycosides) and increased certain sweet-tasting compounds were consistent with the reduced bitter and astringent intensities but enhanced sweet and mellow taste of yellow tea under long-term yellowing treatment.

Volatiles importantly contribute to the odor of yellow tea. Amino acids are also important precursors of volatiles [17]. In our study, the sweet and over-oxidation aroma-related contributors such as organic acids (3-methyl-butanoic acid and n-decanoic acid), ketones (furaneol) and hydrocarbons (1,1-diethoxyethane and 2-ethoxy-propane) were increased after yellowing (Figure 6B), which could be mainly attributed to the Maillard reaction of amino acids and carbohydrates [18,19,20,21]. It was reported that caramel-like odorant furaneol is not only generated from Maillard reaction but also the hydrolysis product of bound furaneol [21]. In addition to amino acids/carbohydrates, lipids, carotenoids and glycosides are also important precursors of odorants [17,18,22]. Lipid oxidation/peroxidation leads to the generation of short fatty acids, alcohols, esters, hydrocarbons and ketones [23,24]. In the present study, acids generated from lipids, such as 2-hexenoic acid and pentanoic acid, were increased with yellowing duration (Figure 6B), among which 2- hexenoic acid with fatty and herbal odors was reported as an oxidation product of hexanal in the heating process [23]. Hexadecanoic acid methyl ester and methyl jasmonate were closely associated with sweet aroma and over-oxidation odor (Figure 5B and Figure 6B), which was thought to be derived from the autooxidation of free fatty acids during the tea manufacturing process, contributing to fruity and flower-like aromas [17,25,26]. Ketone acetophenone, a peroxidation product of lipids with sweet, floral and marzipan flavors [23], was detected in Y2, Y4 and Y7 (Figure 6B) and correlated with sweet aroma in our study (Figure 5B). The above results indicated that the volatiles generated by oxidation of lipids under the high temperature of yellowing treatment were the main components of yellow-tea aroma. The degradation products of carotenoids, such as 2-methyl-4-(2,6,6-trimethyl-1-cyclohexenyl)-3-butenal (α-methyl ional), [4-(2,2,6-trimethyl-7-oxabicyclo[4.1.0]hept-1-yl)]-3-buten-2-one (β-ionone epoxide), megastigmatrienone, α-cadinol and 7,9-Di-tert-butyl-1-oxaspiro(4,5)deca-6,9-diene-2,8-dione, increased with yellowing duration (Figure 6B), which were associated with sweet and over-oxidation aroma of yellow tea (Figure 5B). These norisoprenoids may be produced due to the non-enzymatic degradation of carotenoids under high temperature and humidity in the process of yellowing [27,28,29]. The hydrolysis of glycosidic precursors also plays an important role in the aroma of yellow tea. Geranylgeraniol associated with sweet aroma was generated from the hydrolysis of chloroplasts, which were present in the chloroplasts as geranylgranyl diphosphate-chlorophyllide [30]. Our result showed that *trans*-geranylgeraniol was highly accumulated in Y5 and Y6, suggesting the occurrence of hydrolysis reactions of aroma precursors in the process of yellowing. 

## 4. Materials and Methods

### 4.1. Preparation of Yellow Tea Samples

Yellow tea samples were produced in Shiyi Tea Farm, which is located on Mogan Mountain in Deqing, Zhejiang, China (119°88′ E–119°91′ E, 30°56′ N–30°61′ N). Fresh tea leaves from *Camellia sinensis* cv Zhenong 113 were plucked at the same standard of one bud with two leaves in April. The yellow tea manufacturing procedure was conducted by the steps of spreading, fixation, rolling, yellowing and drying according to the National Standard of China (GB/T 39592-2020) as follows: 

Firstly, the fresh leaves were spread indoors at room temperature for 6 h (spreading), then followed by fixation in a roller fixation machine at 300 °C (fixation). As the leaf temperature decreased to room temperature (moderately softened tea leaves), the tea leaves were rolled in a rolling machine for 25 min based on the principle of “slightly–heavily–slightly”. Subsequently, they were submitted to the yellowing process for 13 h at optimal parameters (leaf temperature of 45 ± 2 °C, leaf water content of 37% ± 3% and ambient relative humidity of 80% ± 5%) according to our previous technological study [9]. One hundred grams of the processed teas were sampled at 0, 3, 4.5, 6, 7.5, 10 and 13h during the yellowing process, respectively. Finally, all samples were dried to s moisture content below 6.5% (90 °C, 30 min), and then all samples were stored at −20 °C for further analysis.

### 4.2. Reagents

The references of non-volatile compounds (HPLC grade ≥ 98%) were purchased from Yuanye Bio-Technology Co., Ltd. (Shanghai, China), including catechins (catechin, epicatechin, catechin gallate, epicatechin gallate, gallocatechin, epigallocatechin, gallocatechin gallate and epigallocatechin-3-gallate), alkaloids (caffeine, theobromine and theophylline), flavonol glycosides (quercetin-3-*O*-glucoside, quercetin-3-*O*-galactoside, quercetin-3-*O*-rutinoside, quercetin-3-*O*-rhamnosyl-glucoside, myricetin-3-*O*-glucoside, myricetin-3-*O*-galactoside, myricetin-3-*O*-rhamnoside, kaempferol-3-*O*-glucoside, kaempferol-3-*O*-rutinoside, kaempferol-3-*O*-rhamnosyl-glucoside) and free amino acids (glutamic acid, aspartic acid, asparaginate, serine, glutamine, histidine, tryptophan, glycine, phenylalanine, arginine, theanine, alanine, γ-aminobutyric acid, tyrosine, threonine, valine, isoleucine, leucine and lysine). Acetonitrile and methanol were HPLC grade and were purchased from Sinopharm Chemical Reagent Co., Ltd. (Shanghai, China). The ultra-pure water (>18 MΩ cm) was prepared by Milli-QTM reference system (Merck Millipore, Milford, MA, USA). 

### 4.3. Sensory Evaluation

The sensory flavor of yellow tea samples was evaluated using quantitative descriptive analysis (QDA). A panel of six expert panelists (three females and three males) was recruited for sensory evaluation, who were specialized in tea sensory analysis with over 10 years of work experience on tea sensory evaluation. Tea infusions for evaluation were prepared according to China Nation Standard (GB/T 23776-2018, Methodology for Sensory Evaluation of Tea). Specifically, 3.0 g of each tea sample were brewed with 150 mL boiling water for 5 min, and then tea infusion was filtered for QDA test. The panelists collected the flavor attributes of yellow tea according to Tea vocabulary for sensory evaluation (National Standard of China, GB/T 14487-2017), Food Sensory Analysis Vocabulary [31]. Based on group discussion, six flavor attributes were adopted, including three taste attributes (umami taste, mellow taste and sweet taste) and three aroma attributes (sweet aroma, over-oxidation aroma and green-tea aroma). “Umami” is a delicious taste mainly induced by amino acids, which were trained by the reference of sodium glutamate solution (0.6 mg/mL) [32]. “Mellow” is a comfortable mouthfeel as smooth as silk, which was trained by the perception of touching silk [31]. “Sweet taste” is a basic mouthfeel induced by sugary food, and sucrose solution (5.8 mg/mL) was used as reference [31]. For aroma traits, sweet aroma is associated with the impression of boiled sweet corn. Over-oxidation aroma is a type of aroma close to the odor of overripe fruits. Green-tea aroma refers to the aroma of curly green tea brewed by boiling water. All the panelists were trained by the references as above to standardize the perception and evaluation of flavor attributes. Each panelist scored the intensity of each attribute on a nine-point scale, ranging from 0 (absent) to 9 (extremely strong). The sensory radar diagram was plotted with the average score of each sensory flavor attribute including taste attributes and aroma attributes [33]. 

### 4.4. Determination of Non-Volatile Compounds 

*Tea infusion preparation:* Each dried yellow tea sample was ground using a mixer mill (FW100, Taisite Instrument Co., LTD, Tianjing, China), then sifted through a 450 μM sieve. The tea powder (0.15 g) was extracted with 25 mL of 50% aqueous ethanol at 70 °C for 30 min, and the mixture was centrifuged at 12,000 rpm and 4 °C for 15 min (3K15, Sigma-Aldrich, Milwaukee, WI, USA). The supernatants were collected for non-volatile compound analysis.

*Determination of catechins and alkaloids:* Catechins and alkaloids (caffeine, theobromine and theophylline) were determined by HPLC with an SPD ultraviolet detector (LC-20A, Shimadzu, Tokyo, Japan) according to a previous study [34]. The HPLC conditions were as follows: Agilent TC–C_18_ column (4.6 × 250 mm, 5 μm, Agilent Technologies, Inc., Santa Clara, California, USA), column temperature 35 °C, mobile phase A = 1‰ formic acid +999‰ water (*v*/*v*) and mobile phase B = 1‰ formic acid + 999‰ acetonitrile (*v*/*v*), the linear gradient elution was as follows: Phase B increased from 20% to 40% during the early 45 min, and then maintained at 20% for 10 min for re-equilibrium. Injection volume was 10 μL, flow rate was 1.0 mL/min and the detection was at 360 nm.

*Determination of flavones and flavonol glycosides.* Flavones and flavonol glycosides were determined by UHPLC-DAD-MS/MS (Waters Corporation, Milford, MA, USA) according to the modified method from a previous study [35]. The chromatographic conditions were as follows: Waters CORTECS T3 column (2.1 × 100 mm, 1.6 μm, Waters Corporation, Milford, MA, USA), column temperature 25 °C, mobile phase A = 0.1% formic acid + 99.9% water (*v*/*v*) and mobile phase B = 100% acetonitrile (*v*/*v*). Gradient change of mobile phase B: 0–1 min, 0.2%; 1–2 min, 0.2%–10.8%; 2–5 min, 10.8%–15.7%; 5–9 min, 15.7%; 9–11 min, 15.7–16.0%; 11–12 min, 16.0%–16.5%; 12–18 min, 16.5%–18.3%; 18–20 min, 18.3%–60.0%; 20–20.01 min, 60.0%–0.2%; 20.01–21 min, 0.2%. Injection volume was 4 μL and flow rate was 0.15 mL/min. Mass spectrometry (MS) was carried out by electrospray ionization (ESI) technique in a negative ion mode. The ion source conditions were as follows: capillary voltage 3 kV, cone voltage 30 V, extractor 3.0 V, RF lens 0.2 V, ion source temperature 150 °C, desolvation gas nitrogen at a flow rate of 400 L/h and temperature 350 °C. Full scans ranging from 200 to 1000 amu were recorded. The wavelengths from 190 to 400 nm were detected by photodiode array detector (DAD), among which 360 nm was used for quantitative determination of flavones and flavonol glycosides. The flavonol glycosides without references were quantified by standard curve conversion of their corresponding mono-glycosides.

*Determination of amino acids.* The determination of amino acids was performed on HPLC with a fluorescence detector based on the pre-column derivatization method with O-phthalaldehyde (OPA) and fluolenylmeghyl chloroformate (FMOC), which was modified accord to previous study [36]. The chromatographic conditions were as follows: Zorbax Eclipse-AAA column (4.6 × 150 mm, 3.5 μm, Agilent Technologies, Inc., Santa Clara, CA, USA), column temperature 40 °C, mobile phase A = Na_2_HPO_4_ aqueous solution (40 mM, pH 7.8) and mobile phase B = 45% acetonitrile + 45% methanol + 10% water (*v*/*v*), injection volume 10 μL. Linear gradient change of mobile phase B: 0–18 min, 5%–60%; 18–23 min, 60%–100%; 23–30 min, 5%. The flow rate was 1.5 mL/min and the detection at 340 nm (excitation wavelength) and 450 nm (emission wavelength).

*Determination of soluble sugars.* The content of soluble sugars was determined by anthraquinone colorimetric method based on a previous study [37], with some modifications. The standard curve of glucose solution was used for quantitative analysis of soluble sugars in tea infusion. Tea infusion (100 μL) was mixed with 400 μL of water and 4 mL of anthrone sulfuric acid solution, and the mixture was placed in a boiling water bath for 7 min. After cooling at room temperature for 10 min, the absorption value of the mixture was determined by ultraviolet spectrophotometer at 620 nm. The soluble sugars were quantified as mg glucose equivalent/g dry weight.

### 4.5. Determination of Volatiles 

*Extraction of volatiles.* Simultaneous distillation and extraction (SDE) method was used for volatile extraction based on the modified method [38]. A steam generator was under a cylindrical tea container, both of which were connected by a glass delivery tube. The tea container and extraction solvent evaporator were respectively connected with two sides of a condenser. Fifteen grams of tea sample were placed into the tea container. In total, 250 mL of water and 30 mL of ethyl ether (containing 1 ppm internal standard butylated hydroxytoluene (BHT)) were injected into a water steam generator and extraction solvent evaporator, respectively. The water steam generator and extraction solvent evaporator were, respectively, kept at 100 and 40 °C for continuous evaporation of water and ethyl ether solution. Tea volatiles carried by water steam were extracted by the ether steam in the extraction chamber, and then were condensed by a water-reflux condenser. After extraction for 120 min, the collected volatiles-ether extract was dehydrated with 5 g anhydrous sodium sulfate overnight, and then was concentrated to 1 mL. 

*Analysis of volatiles by gas chromatography–mass spectrometry (GC-MS).* GC-MS analysis of volatiles was performed on Shimadzu gas chromatograph 2010-plus with triple quadruple mass spectrometer QP 2020 (Shimadzu, Tokyo, Japan), using the conditions based on a previous study [39]. The GC conditions were as follows: SH-Rxi-5Sil MS capillary column (0.25 mm × 30 m, 0.25 μm, Shimadzu, Tokyo, Japan), injection port temperature 250 °C and injection mode was splitless. Carrier gas was high purity helium (>99.999%) and flow rate was 1 mL/min. Linear gradient change of oven temperature: 0–5 min, 50 °C; 5–53.5 min, 50 °C–210 °C; 53.5–58.5 min, 210 °C; 58.5–60.0 min, 210 °C–230 °C; 60.0–65.0 min, 230 °C. Mass spectrometer conditions: ion source temperature 250 °C, electron energy 70 eV, solvent delay time 3 min and full scan range from 35 to 450 amu. The raw data was deconvolved by LabSolutions GC-MS solution (4.45 SP1, Shimadzu, Tokyo, Japan) firstly, and then all the compounds were filtrated by retain frequency >60% and variability coefficient < 25%, and then were identified by mass spectra comparison with NIST 17 library (similarity degree > 70). The identified volatiles were quantified by an internal standard BHT as following [40]:

$$\mathrm{Peak}\;\mathrm{area}\;\mathrm{ratio}\;\left(\mathrm{PAR}\right)=\frac{\mathrm{Peak}\;\mathrm{area}\;\mathrm{of}\;\mathrm{target}}{\mathrm{Peak}\;\mathrm{area}\;\mathrm{of}\;\mathrm{BHT}}$$

### 4.6. Statistical Analysis

All analyses were replicated thrice. Variance analysis (ANOVA) was performed on SPSS software (v21.0, SPSS Inc., Chicago, IL, USA). The orthogonal partial least squares discrimination analysis (OPLS-DA) was carried out by SIMCA 14.1 (Umetrics AB, Umea, Sweden). Multiple factor analysis (MFA) and multidimensional alignment (MDA) were used as the datasets integration analysis method to explore the relationships between sensory flavor traits and key non-volatile/volatile compounds, using XLSTAT 2019 (Addinsoft, New York, NY, USA).

## 5. Conclusions

This study investigated the effect of yellowing duration on the non-volatile and volatile profiles of yellow tea and the associations with sensory traits. The intensities of umami and green-tea aroma were reduced, sweet taste, mellow taste and sweet aroma were increased with yellowing time, while the over-oxidation aroma of yellow tea was produced under long-term yellowing treatment. A total of 54 non-volatiles and 176 volatiles were determined in the 21 yellow tea samples under 0–13 h yellowing treatments, among which 67 compounds were identified as the key chemical compounds correlated with sensory traits, mainly including catechins, gallic acid, flavones and flavonol glycosideses, caffeine, amino acids, ketones, acids, alcohols, esters, hydrocarbons, aldehydes, phenolic compounds, pyrroles and sulfocompounds. The increase in sweet and mellow tastes during yellowing was attributed to the increase in gallic acid and sweet amino acids (e.g., serine, tyrosine threonine and alanine), with the assistance of the reductions in bitter and astringent matters like catechins, caffeine, flavones and flavonol glycosides. The elevated sweet aroma was related to the increase in sweet, herbal and woody odorants in yellow teas, while the over-oxidation aroma was attributed to the increase in fatty and fermented odorants, mainly due to the non-enzymatic reactions like degradation, oxidation and hydrolysis under thermal and high humidity conditions. This study reveals the chemical changes underlying the yellowing process and their associations with sensory traits, which provides a chemistry foundation for further improving the quality of yellow tea.

## Data Availability

The data are available to the researches upon request.

## Supplementary Materials

The supplementary material is available online. Table S1: Sensory-traits values of yellow teas with different yellowing time.

## Sample Availability

Samples of the compounds are not available from the authors.
