# Supplementary material for: Effect of Yellowing Duration on the Chemical Profile of Yellow Tea and the Associations with Sensory Traits
Source: Molecules. 2022 Jan 29;27(3):940. doi: 10.3390/molecules27030940 (PMC8839223; doi:10.3390/molecules27030940)
Supplement: Supplementary file 1 [file molecules-27-00940-s001.zip › molecules-1535979-supplementary.pdf]

Table S1 Sensory-traits values of yellow teas with different yellowing time

Sensory traits	Y1	Y2	Y3	Y4	Y5	Y6	Y7
Sweet flavor	0.50 ± 0.55^{e}	2.50 ± 0.55^{d}	5.50 ± 0.55^{c}	7.08 ± 0.49^{b}	8.17 ± 0.41^{a}	9.00 ± 0.00^{a}	7.00 ± 0.32^{b}
Over-oxidation	0.00 ± 0.00^{c}	0.00 ± 0.00^{c}	0.00 ± 0.00^{c}	0.00 ± 0.00^{c}	0.00 ± 0.00^{c}	2.50 ± 0.55^{b}	4.58 ± 0.66^{a}
Green-tea aroma	8.83 ± 0.41^{a}	6.33 ± 0.52^{b}	3.83 ± 0.75^{c}	1.50 ± 0.55^{d}	0.17 ± 0.41^{e}	0.00 ± 0.00^{e}	0.00 ± 0.00^{e}
Umami	8.67 ± 0.52^{a}	6.33 ± 0.52^{b}	4.50 ± 0.55^{c}	2.67 ± 0.52^{d}	1.50 ± 0.55^{d}	0.17 ± 0.41^{e}	0.00 ± 0.00^{e}
Softness	0.50 ± 0.55^{e}	1.83 ± 0.41^{d}	3.67 ± 0.52^{c}	5.50 ± 0.55^{b}	6.83 ± 0.75^{b}	8.17 ± 0.75^{a}	8.67 ± 0.52^{a}
Sweetness	0.33 ± 0.52^{d}	1.50 ± 0.55^{d}	3.50 ± 0.55^{c}	5.67 ± 0.52^{b}	7.00 ± 0.63^{ab}	8.00 ± 0.63^{a}	6.17 ± 0.75^{b}

Data with different alphabetic letters in a same row were significantly different at $p = 0.05$.
